# Characterization and Vibro-Acoustic Modeling of Wood Composite Panels

**DOI:** 10.3390/ma13081897

**Published:** 2020-04-17

**Authors:** Andrea Santoni, Paolo Bonfiglio, Patrizio Fausti, Cristina Marescotti, Valentina Mazzanti, Francesco Pompoli

**Affiliations:** 1 Engineering Department, University of Ferrara, 44122 Ferrara, Italy; patrizio.fausti@unife.it (P.F.); cristina.marescotti@unife.it (C.M.); valentina.mazzanti@unife.it (V.M.); francesco.pompoli@unife.it (F.P.); 2 Materiacustica srl, 44122 Ferrara, Italy; paolo.bonfiglio@materiacustica.it

**Keywords:** wood plastic composite, transmission loss, radiation efficiency, orthotropic panel, wavenumber analysis

## Abstract

Natural fiber-filled polymers offer good mechanical properties and economic competitiveness compared to traditional materials. Wood flour is one of the most widely used fillers, and the resulting material, known as wood plastic composite (WPC), has already found a wide applicability in many industrial sectors including automotive and building construction. This paper, as a followup of a previous study on a numerical-based approach to optimize the sound transmission loss of WPC panels, presents an extensive numerical and experimental vibro-acoustic analysis of an orthotropic panel made out of WPC boards. Both structural and acoustical excitations were considered. The panel radiation efficiency and its transmission loss were modeled using analytic and semi-analytic approaches. The mechanical properties of the structure, required as input data in the prediction models, were numerically determined in terms of wavenumbers by means of finite element simulations, and experimentally verified. The accuracy of the predicted acoustic performances was assessed by comparing the numerical results with the measured data. The comparisons highlighted a significant influence of the junctions between the WPC boards, especially on the panel’s transmission loss. The radiation efficiency results were mostly influenced by the boundary conditions of the plate-like structure. This latter aspect was further investigated through a finite element analysis.

## 1. Introduction

The building construction industry is responsible for a significant amount of CO_2_ global emissions and energy consumption. Taking climate actions within this sector can be very effective, even though, as shown in the 2019 Global Status Report on buildings and construction [1], the final energy demand in buildings is still rising. The strategic actions that should be undertaken in order to pursue the decarbonization of the building and construction industry span from increasing the use of renewable energy sources, to installing more efficient heating, cooling, and ventilation systems, but it also involves the development of innovative materials or bio-based solutions with a reduced impact on the environment. In order to be competitive against traditional systems, such innovative solutions need to be optimized both in terms of performances, as well as in their manufacturing process, other than being economically sustainable. The use of thermal and acoustical insulating systems involving natural or recycled materials is only at an early stage and still limited, as depicted by the state-of-the-art published a few years ago [2]. Nevertheless, natural fibers may represent a relatively inexpensive and valuable alternative to traditional synthetic thermal or sound insulation materials [3]. In fact, as was shown in a recently published paper [4], with an appropriate manufacturing process, it is possible to obtain natural hemp-based fibrous materials with a sound absorption coefficient comparable to the one provided by traditional synthetic fibrous materials. Besides, natural fiber-filled polymers offer both good mechanical properties and economic competitiveness compared to traditional materials. Among natural fibers, wood flour is one of the most widely used fillers, mainly because of its wider availability. The resulting material is usually known as wood plastic composite (WPC) [5]. The optimum properties are obtained using a wood fiber filling level of about 50 wt.%, the fibers possessing an aspect ratio of 10 or higher and using an amount of coupling agent around 4 wt.% [6,7]. As found in recent studies [8,9], the use of polypropylene as the matrix would improve the mechanical properties of WPC systems. However, it would also result in a more difficult processing and characterization [10,11]. In fact, the use of polyethylene allows for a lower processing temperature, reducing the risk of degrading the wood fiber [12]. WPC boards and panels are already widely involved in external flooring and decking, thanks to a better outdoor durability also in a wet environment compared to natural wood. WPC components also find wide application in the automotive industry [13]. Moreover, WPC systems can also be used in building construction applications [14] such as roofing systems, walls, and façades, or again as sound barriers. Due to the increasing interest in such a composite material, in the last few years, several studies have been published, investigating for example the influence of different wood fibers or of different types of matrix [15] or proposing the optimization and improvement of the mechanical or thermal properties of WPC systems [16,17].

As a followup to a previous study [18], which proposed a numerically-based approach to optimize the sound insulation performance of WPC panels, this paper presents an extensive numerical and experimental vibro-acoustic analysis of an analogous structure, increasing the knowledge on its dynamic response to a structural or acoustic excitation. The structural wavenumbers obtained from finite element (FE) simulations were used as input data for analytical and semi-analytical computational models to predict the panel radiation efficiency and its transmission loss (TL). The accuracy of the predicted results was assessed by comparing the numerical and measured data. The aim of this work is not limited to the presentation of computationally efficient approaches to investigate orthotropic plate-like structures, but especially to highlight and explain the discrepancies that are likely to be found between the predicted performance and the one observed in real structures. In the next section, the considered WPC element is described. In the same section, the numerical models implemented to characterize the elastic properties of the structure and to investigate its acoustic performance, both in terms of radiation efficiency and TL, are briefly introduced. Section 3 describes the experimental setup and the measurements performed on a WPC panel. Numerical results are presented, discussed, and validated, by comparison with experimental data, in Section 4.

## 2. Materials and Methods

**Figure 1:**
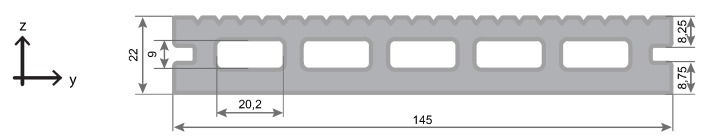
Cross-section of the tested wood plastic composite (WPC) boards. Dimensions are given in mm.

The test panel was made of extruded WPC boards of high density polyethylene (HDPE) filled with 50 wt.% of wood fibers from pine sawdust, with a density ρ=1316$\mathrm{kg}/m^{3}$, manufactured by Iperwood srl (Ferrara, Italy). The material’s static elastic modulus, E=5.4 GPa, was determined from the frequency response function (FRF) of a clamped-free homogeneous bar of the WPC material, excited by an impact hammer [18], by means of a resonant approach based on Oberst’s beam method [19]. The geometry of the 22 × 145 ${\mathrm{mm}}^{2}$ cross-section of the tested hollow boards is shown in Figure 1. These extruded boards were used to build a WPC panel, as clearly explained in the following part of the paper.

### 2.1. Characterization of the Panel’s Elastic Properties

The bending stiffness of a plate-like structure is required as input data in several vibro-acoustic models. For the considered WPC components, this was investigated in terms of the wavenumbers of the structural waves, by means of a well-established wave-correlation approach. This required the evaluation of the dynamic response of the tested structure, usually due to a broadband excitation, along a line or over a grid of equally distributed points. This method was implemented in the form known as inhomogeneous wave correlation (IWC) [20,21,22]. A correlation function between a mono-dimensional inhomogeneous plane propagating wave, defined as $o\left(k_{x}\right)=\exp\left(-jk_{x,r}+k_{x,i}\right)x$, and the vibrational field evaluated on the structure $\tilde{w}\left(\omega ,x_{i}\right)$, can be expressed as:

(1) $$F\left(\omega ,k_{x}\right)=\frac{\sum_{i}\tilde{w}\left(\omega ,x_{i}\right)o^{*}\left(k_{x}\right)\Delta x}{\sqrt{\sum_{n}{\left|\tilde{w}\left(\omega ,x_{i}\right)\right|}^{2}\Delta x\sum_{n}{\left|o^{*}\left(k_{x}\right)\right|}^{2}\Delta x}}$$

where ω is the angular frequency, Δx represents the spacing expressed in meters between two adjacent evaluation positions, while $k_{x,r}$ and $k_{x,i}$ represent the real and the imaginary components of the complex wavenumber $k_{x}$, the symbol ${}^{*}$ is used to indicate the complex conjugate, and j indicates the imaginary unit. The wavenumber dispersion relation is identified by maximizing the function $F\left(\omega ,k_{x}\right)$ given in Equation (1). The wave-correlation-based approaches allows investigating elastic and viscoelastic materials in a broad frequency range [23,24,25,26]. Moreover, it could be convenient to fit the evaluated wavenumbers with an analytical dispersion relation. In this case, the one derived form Mindlin’s theory for thick plates [27] was used:

(2) $$k^{4}-\left[k_{L}^{2}+{\left(\frac{k_{T}}{\kappa }\right)}^{2}\right]k^{2}-k_{B}^{4}+\frac{{\left(k_{L}k_{T}\right)}^{2}}{\kappa^{2}}=0$$

where $k_{L}$, $k_{T}$, and $k_{B}$ represent the wavenumbers for longitudinal, transverse, and pure bending waves, respectively:

(3) $$\begin{array}{ccc} k_{L}=\omega \sqrt{\frac{\rho \left(1-\nu^{2}\right)}{E}};\;\; & k_{T}=\omega \sqrt{\frac{\rho }{G}};\; & \;\;k_{B}=\sqrt{\omega }\sqrt[{4}]{\frac{\mu }{D}} \end{array}$$

The apparent elastic properties of the structure, *E* and *G*, are the fitting variable; ν represents the structure’s Poisson ratio, *D* its effective bending stiffness, and ρ and μ its equivalent density and mass per unit of area, respectively. The coefficient κ, introduced to account for the non-uniform distribution of shear stress over the cross-section, can be computed from Poisson’s ratio ν, as described in [28,29].

**Figure 2:**
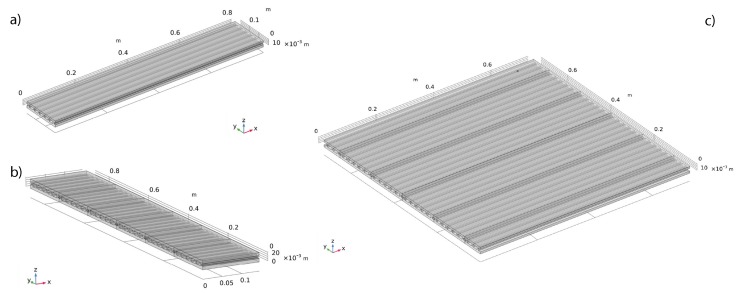
FE model of the tested WPC components: (**a**) x-wise wave propagation analysis: model of an extruded board; (**b**) y-wise wave propagation analysis: model of sections of boards coupled together; (**c**) model of the considered plate.

**Figure 3:**
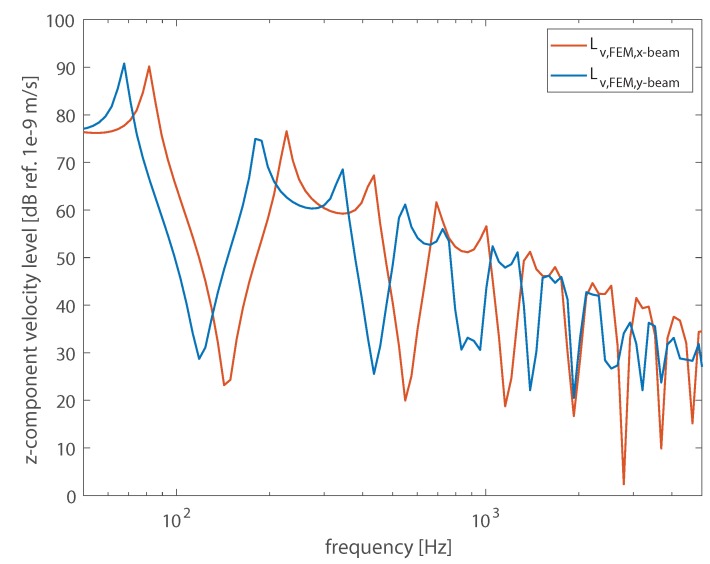
Vibration velocity levels of the vertical component of the vibration velocity computed from the FE models and averaged over the beams’ surface.

The dynamic response of the WPC components was initially investigated by modeling an extruded beam in an FE framework, by using the software COMSOL Multiphysics^®^, as shown in Figure 2a). WPC panels are constituted of beams assembled and joined together. Due to their geometric configuration, these panels may exhibit an orthotropic behavior. Therefore, it was necessary to also investigate the orthogonal direction, in which a plate would be made by laterally-jointed sections of extruded boards, as shown in Figure 2b). The junction between two adjoined boards was simplified by imposing a continuity condition at the interface surface. The 3D models for the considered beams were meshed ensuring that the maximum element size was ten times smaller than the wavelength associated with the highest investigated frequency. The beams were excited by a broadband boundary load applied in the vertical direction, as shown in Figure 3 by the velocity levels of the vertical component. The vibration field was evaluated over a line of points, evenly spaced 2 cm from one another.

### 2.2. Plate’s Radiation Efficiency

The real part of the radiation impedance of a vibrating structure, normalized with respect to the acoustic impedance, represents its radiation efficiency σ. This is a non-dimensional vibro-acoustic descriptor that quantifies the capability of the structure to convert vibrational energy into acoustic energy as sound waves. It is particularly convenient to evaluate such a descriptor in order to assess the acoustic performance of a vibrating structure excited by mechanical sources. The radiation efficiency of the considered orthotropic WPC panel was computed by means of two different approaches. Firstly, the radiation efficiency was computed with a modal approach assuming the WPC panel as an equivalent thin orthotropic panel with simply supported boundaries and inserted into a infinite rigid baffle. The effect of fluid load was neglected. These very same assumptions on the plate configuration were also used to evaluate the modal-average radiation efficiency, considering a high modal density within the entire investigated frequency range. Both models take into account the orthotropic behavior of the WPC panel by means of a direction-dependent bending stiffness, which is defined as:

(4) $$D\left(\phi \right)=D_{x}\cos^{4}\phi +2D_{xy}\cos^{2}\phi \sin^{2}\phi +D_{y}\sin^{4}\phi$$

The angle ϕ represents the azimuthal angle of propagation of the structural wave. $D_{x}$ and $D_{y}$ are the bending stiffnesses associated with the plate’s principal directions. $D_{xy}$ is the effective torsional stiffness. In this case, the orthotropic bending stiffness given in Equation (4) was approximated by assuming $D_{xy}\approx \sqrt{D_{x}Dy}$. Thus, it was possible to compute an effective bending stiffness $D_{i}$ along the *i*th principal direction from the wavenumbers $k_{i}$ obtained from numerical models as described in the previous section:

(5) $$D_{i}\left(\omega \right)=\frac{\omega^{2}\mu_{i}}{k_{i}^{4}\left(\omega \right)};\;\;i=x,y$$

The characterization of the elastic properties of the structure through the effective bending stiffness, given in Equation (5), allows compensating for the effects of shear deformation and rotatory inertia, neglected by the thin plate theory, which, however, may affect the plate’s response at high frequencies. For all the details regarding the implemented equations, please refer to the work of Santoni et al. [30], in which the two models were developed in order to calculate the radiation efficiency of orthotropic cross-laminated timber panels.

It has already been proven that these two models are computationally efficient and provide accurate results for a thin orthotropic plate with simply supported boundaries. However, such a restraint condition is hardly feasible in real structures, which often present more complex boundary conditions. Therefore, we also investigated the radiation efficiency of the WPC panel by means of an FE model, using the software COMSOL Multiphysics^®^, allowing considering different boundary conditions. The plate, with a radiating surface S=0.73×0.73=0.533$m^{2}$, was modeled coupling together five WPC boards by imposing a continuity condition at the interface surfaces. The mesh was generated in order to guarantee that the maximum element size was ten times smaller than the wavelength associated with the highest investigated frequency. A point force was applied in the position with coordinates $x_{f}=0.65$ m and $y_{f}=0.70$ m. The radiation efficiency was computed from the vibration velocity field evaluated over a uniform grid of points, spaced 5 cm from one another, by using a well-established hybrid approach know as the discrete calculation method [31]. This approach, assuming radiation in the free-field, combines the complex vibration velocity, evaluated over the surface of the considered structure, with the analytical computation of the radiation impedances. Therefore, the results obtained for the simply supported plate were directly comparable with the radiation efficiency analytically computed with the modal approach.

### 2.3. Plate’s Transmission Loss

When a structure is excited by an acoustic field rather than a mechanical force, it is convenient to evaluate its acoustic performance in terms of sound transmission loss (TL). Sound transmission through the considered WPC panel was computed by means of the transfer matrix method (TMM) [32], an easily implementable approach with wide-ranging applicability to model wave propagation through laterally infinite media of different natures. The WPC plate was modeled as a single thin orthotropic layer [33,34], characterized by the wave impedance:

(6) $$Z\left(\omega ,\phi \right)=j\omega \mu \left(1-\frac{D\left(\phi \right)k_{t}^{4}}{\omega^{2}\mu }\right),$$

The orthotropic bending stiffness $D\left(\phi \right)$ is given in Equation (4), where $k_{t}$ is the trace wavenumber defined as $k_{t}=k_{0}\sin\theta$, with $k_{0}$ the acoustic wavenumber and θ the angle of incidence of the acoustic plane wave exciting the structure. The structure modeled within the TMM was assumed to be laterally of infinite extent; modal resonances and the diffraction effect caused by the finite dimension of real structures were not considered. Since sound transmission below the first coincidence is governed by forced vibration and modal resonances, such assumption may cause a significant lack of accuracy at low frequencies. However, the results could be improved by considering the non-resonant radiation efficiency of the finite size panel, as described by Villot et al. [35]. Other different formulations have been proposed to compute such a non-resonant radiation efficiency [36,37]; in particular in this case, the one developed by Rhazi et al. [38] was applied. Assuming a random incidence diffuse field excitation, the WPC plate’s sound transmission loss is determined for each angular frequency ω as:

(7) $$TL\left(\omega \right)=-10\log\frac{\int_{0}^{2\pi }\;\;\;\int_{0}^{\pi /2}\tau \left(\omega ,\theta ,\phi \right)\sin\theta \cos\theta \;d\theta d\phi }{\int_{0}^{2\pi }\;\;\;\int_{0}^{\pi /2}\sin\theta \cos\theta \;d\theta d\phi }$$

where $\tau \left(\omega ,\theta ,\phi \right)$ is the sound transmission coefficient computed for each frequency ω with the TMM and eventually corrected with the non-resonant radiation efficiency of the finite size plate. It is also dependent on the angle of incidence of the impinging plane wave θ and the azimuthal angle ϕ.

## 3. Experimental Investigation

**Figure 4:**
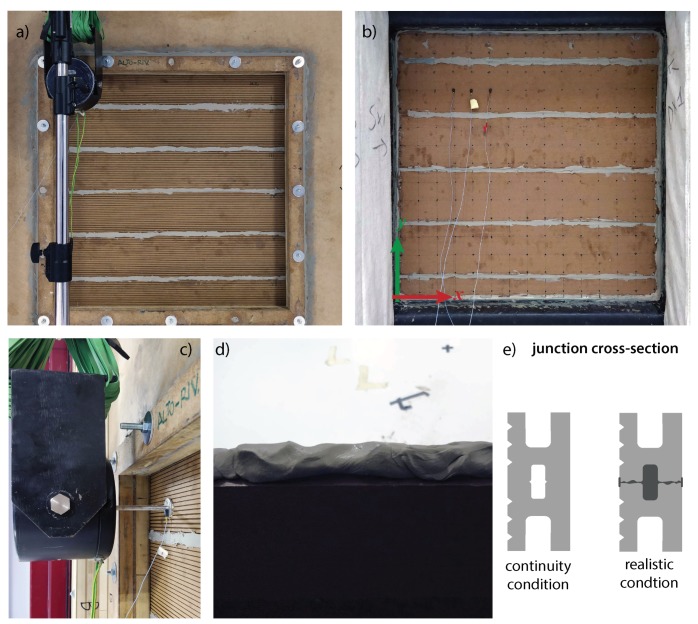
Pictures of the experimental setup used to investigate the WPC response to both a mechanical and an acoustic excitation: (**a**) panel view from the exciting side (reverberant room); (**b**) panel view from the receiving side (semi-anechoic room); (**c**) detail of the mechanical excitation; (**d**) details of the elastic putty layer applied to join the WPC boards vertically; (**e**) digram of the junction between two WPC boards.

In order to validate the results computed from the prediction models and the numerical simulations described in the previous section, a WPC plate was experimentally investigated. A rectangular panel, with a radiating surface with the same dimensions considered in the numerical models: $L_{x}\;=\;0.73$ m, $L_{y}\;=\;0.73$ m and h=0.022 m, was mounted into the testing window between the reverberant room and the semi-anechoic chamber of the University of Ferrara. The plate was realized joining together six WPC boards by means of high-density elastic putty, with a measured density of about $\rho_{putty}=2400$$\mathrm{kg}/m^{3}$. The panel was secured by drilling holes in some of the boards and installing a wood frame fixed with threaded bolts. Since mechanical fixings were not employed to join the WPC beams, a significant amount of elastic putty was used to build the panel. It was applied both on the beams’ junctions, through their entire thickness, and on the panel edges in order to keep the panel in place and especially to prevent any sound leakage. Figure 4 provides some photos of the experimental setup. Moreover, a digram of the junction between two WPC boards is shown in Figure 4e). Even though the presence of the elastic putty was neglected in the numerical model, by assuming a continuity condition between adjacent boards, it certainly influenced the wave propagation. Nevertheless, due to an obvious complexity and to the lack of knowledge of the material properties, it was not possible to quantify these effects accurately. However, as will be shown in the next section, it was possible to evaluate the effect of the additional mass, introduced by the elastic putty, on the panel dynamic response.

The plate was mechanically excited by means of an electro-dynamic shaker B&K Type 4809 driven with a white noise signal. The shaker was suspended with rubber bands on the reverberant room side and rigidly fixed by gluing the stinger termination to the WPC plate, in the position $x_{f}=0.65$ m and $y_{f}=0.70$ m, according to the system shown in Figure 4, in order to replicate the numerical model. To evaluate the structural wavenumbers propagating along the two principal directions experimentally, the electro-dynamic shaker was driven with a broadband sine-sweep signal, from 50 Hz to 6000 Hz. The vibrational field was measured by means of miniature accelerometers PCB 352C22 with a sensitivity of 10 mV/g, along two orthogonal lines of points, evenly spaced 2 cm from one another, aligned with the excitation position. The impulse response of each measured point with respect to the reference signal was determined by means of the convolution technique. More details on this experimental approach can be found in [25]. Moreover, the vibration field on the panel’s surface, due to a structure-borne white noise excitation within the frequency range 50 Hz–10 kHz, was also measured over a grid of points with a spacing of 5 cm, in order to evaluate the radiation efficiency of the WPC plate by means of the discrete calculation method, already introduced in the previous section. So as to have a constant phase relationship between non-simultaneous measurement, a reference transducer was placed on the shaker stinger termination. The vibration velocity was derived by dividing the measured acceleration by the factor jω, within the frequency domain.

In the reverberant room, which had a volume of 250.7 $m^{3}$, three omnidirectional sound sources were separately driven by a unrelated stationary white noise, and the average sound pressure level $L_{p}$ was measured using six microphones placed in different positions. The receiving room had highly absorbing lateral walls and ceiling and a reflective floor. The average sound intensity $L_{i}$ was measured inside this room at a distance of approximately 10–15 cm from the panel, by a manual scanning procedure using a B&K 3547 sound intensity probe. The plate’s sound transmission loss was determined from such quantities according to the ISO 15186-1:2003 Standard [39].

## 4. Results

**Figure 5:**
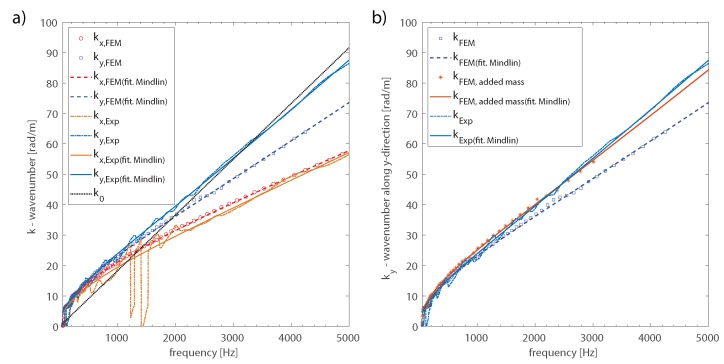
Wavenumbers determined numerically (FEM) and experimentally (Exp). (**a**) Comparison between the structural wavenumbers along the principal directions of the orthotropic WPC panel and the acoustic wavenumber; (**b**) wavenumbers determined along the *y*-direction considering the additional mass of the elastic putty.

A first comparison was made between the wavenumbers evaluated from the FE beam models, introduced in Section 2, and the experimental data measured on the WPC plate, as described in the previous section. Figure 5a) presents the wavenumbers, along the principal directions *x* and *y*, directly determined by maximizing the inhomogeneous wave correlation function, given in Equation (1), and the fitted curve obtained using Mindlin’s dispersion relation given in Equation (2). A rather good agreement was found between the numerical and experimental wavenumbers determined along the *x*-direction. On the other hand, above 2000 Hz, the experimental wavenumber measured along the *y*-direction was significantly higher than the wavenumber computed from the FE model. According to the results determined from the FE model, along the *x*-direction, the first coincidence frequency fell between 1100 Hz and 1400 Hz, consistent with what was found from the experimental wavenumbers. While along the *y*-direction, the first coincidence frequency was found around 1900 Hz from the numerical model, it shifted up to approximately 3000 Hz according to the experimental wavenumbers measured on the WPC plate. The increase of the structural wavenumber, which corresponded to a reduction of the bending stiffness, was caused by the mass added to the structure by the elastic putty, which was not considered in the numerical model. The presence of this material, used to join the WPC boards vertically, strongly affected the *y*-direction of propagation, while its influence was negligible along the *x*-direction. In fact, as shown in Figure 5b), the structural wavenumbers obtained along the y-direction from the FE model by considering the additional mass of the elastic putty were in rather good agreement with the experimental results.

**Figure 6:**
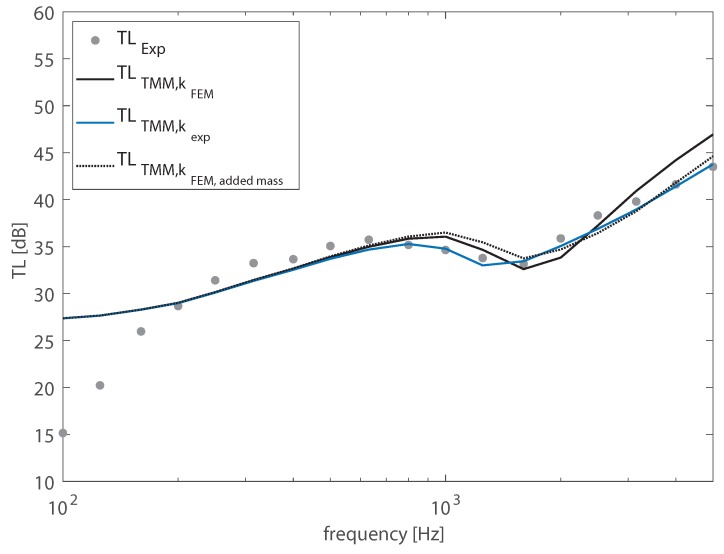
Transmission loss (TL) of the WPC sandwich plate in 1/3-octave bands. Comparison between the results computed by means of the transfer matrix method (TMM) using as input data the structural wavenumber $TL_{TMM,k_{j}}$ and the experimental data $TL_{Exp}$.

It should be noted that the way in which the boards were coupled in order to realize a plate-like structure was suitable for laboratory conditions, but it would never be implemented in practical structures, for which the junctions need to be properly and conveniently designed. However, it is interesting to evaluate how the discrepancies observed between the numerical and the experimental wavenumbers, when the junctions were not considered, may affect the prediction of the acoustic performance of the structure. Therefore, the radiation efficiency and the transmission loss of the WPC panel computed using as input data the numerical wavenumbers, obtained from the FE models, were compared both with the results computed from the experimental wavenumbers and with the radiation efficiency and the TL measured in the laboratory. As shown in Figure 6, the TMM provided a good approximation of the experimental TL, except in the lowest frequency bands, were the panel response was governed by structural modes, which were not considered in the TMM framework. At the higher frequencies, using the experimental wavenumbers as input data certainly offered a better accuracy. In fact, compared to the results computed from the FE-based wavenumber, the coincidence region was better represented. The mass added to the system tended to reduce the stiffness above 2000 Hz. As already mentioned, this shifted the first coincidence towards higher frequencies. Moreover, it allowed better approximating the TL above the critical frequency. In fact, the experimental wavenumber increased with the frequency at a higher rate, with values that were close to the acoustic wavenumber in a wide range of frequencies. For this reason, the experimental wavenumbers allowed for a better prediction of the plate’s TL, which exhibited a wider coincidence region. Figure 6 also reports the TL computed using the wavenumbers determined from the FE model in which the added mass was considered along the *y*-direction, obtaining a greater accuracy above the critical frequency.

**Figure 7:**
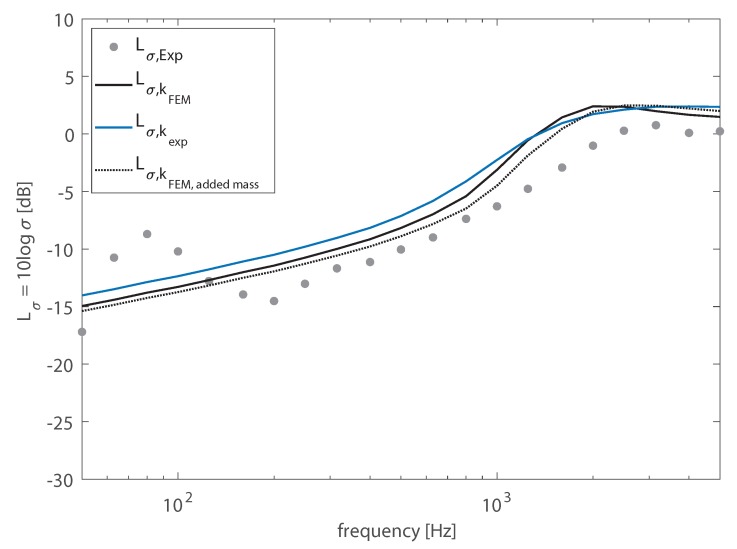
Radiation ratio of the WPC panel in 1/3-octave bands. Comparison between the results computed using as input data the structural wavenumber, determined from FE model $L_{\sigma ,k_{FEM}}$, or experimentally $L_{\sigma ,k_{exp}}$, and the experimental data $L_{\sigma ,Exp}$.

An analogous comparison was made between the radiation efficiency measured in the laboratory and the modal-average radiation efficiency computed from numerical and experimental wavenumbers, as reported in Figure 7. Even though some of the effects described for the plate’s TL also characterized sound radiation, the comparison in terms of radiation ratio $L_{\sigma }=10\log\sigma$, given in Figure 7, highlighted more severe discrepancies between computed and numerical results. In fact, the effect of the added mass was limited to a wider and a less sharp peak associated with the coincidence region, obtained from the numerical wavenumbers. A slightly better accuracy could be obtained by taking into account the effect of the added mass in the numerical model employed to determine the structural wavenumbers, even though a significant deviation could still be observed. Thus, the effect of the mass added by the elastic putty along the junctions did not explain why above 2000 Hz, a substantially lower radiation ratio was experimentally determined. Both radiation efficiency models, introduced in Section 2, assumed simply supported boundary conditions. Although this assumption allowed for a computationally efficient model, such a condition is difficult to realize in practice. The boundary conditions of the tested WPC panel were rather complex and difficult to reproduce with a numerical model. However, it was possible to investigate their influence on sound radiation analyzing the radiation efficiency obtained by means of the DCMfrom the vibration velocity distribution evaluated using an FE model of the plate with several boundary conditions: SS-SS indicates that all the edges are simply supported (this condition is directly comparable with the modal-based analytic formulation); FF-FF indicates that all the edges are free; SS-FF indicates that the left and right edges are simply supported and the top and bottom edges are free; FF-SS indicates that the left and right edges are free and the top and bottom edges are simply supported.

**Figure 8:**
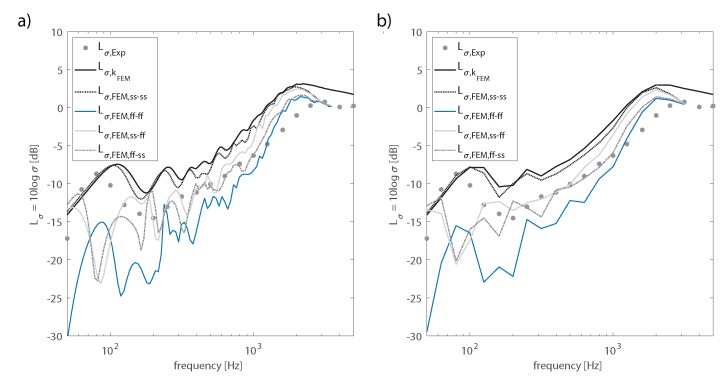
Influence of the boundary condition of the radiation ratio. Comparison between the modal-based analytical approach and the data obtained from FE simulations. (**a**) Radiation ratio narrow frequency bands; (**b**) radiation ratio 1/3-octave bands.

As shown both in narrow frequencies and in one-third octave bands in Figure 8, the radiation ratio obtained from the FE model with simply supported boundary conditions $L_{\sigma ,FEM,ss-ss}$ was in good agreement with the analytical model fed with the numerical structural wavenumbers as input data $L_{\sigma ,k_{FEM}}$. The peak exhibited by the ration ratio at the lowest frequencies seemed to indicate that the simply supported condition was a good approximation of the edge fixing of the installed plate. However, as the frequency increased, the experimental radiation ratio was better approximated by the free-edge condition or by a combination of free and simply supported edges. In fact, as already found in other studies [40], decreasing the degree of restraint at the edges reduced the radiation efficiency of the panel. The restraint at the plate’s edges was provided by a wood frame fixed with threaded bolts. Strips of a resilient viscoelastic material were interposed between the wood frame and the WPC panel along its perimeter, to prevent sound leakages. Results showed that, at low frequencies, the cross-section of the plate at the border was able to rotate, but the horizontal motion was prevented. However, as the frequency increased, the restraint to the horizontal motion provided by the experimental setup decreased, allowing, at least to some extent, the cross-section of the beam to move back and forth.

## 5. Conclusions

This paper presented a vibro-acoustic analysis of a WPC orthotropic panel, in which the predicted results were compared with measured data. The sound transmission loss and the radiation efficiency of a WPC panel were computed by means of analytical and semi-analytical models. The input data for these models were the structural wavenumbers associated with the plate’s principal directions, obtained from FE simulations. A first validation, performed comparing the numerical wavenumbers with experimental results, highlighted a significant deviation along the direction over which the extruded boards were joined. In fact, the additional mass introduced onto the plate surface by the high-density elastic putty used to join the WPC boards strongly affected wave propagation along the vertical direction, emphasizing the orthotropic behavior of the panel. Even though the predicted TL approximated with satisfying accuracy the general trend of the experimental data, it was found that a better accuracy could be reached by using as input data the experimental TL, taking into account the effects of the added mass. These findings highlighted the importance of a proper design of the junction systems of real plate-like WPC structures; a convenient optimization of these joints might represent a possibility to further increase the vibro-acoustic performance of the system. The radiation efficiency obtained from analytical models was found to overestimate the experimental results. By means of an FE analysis, it was shown that the deviation between the predicted and measured radiation efficiency was caused by the real boundary conditions of the WPC panel, which were not simply supported as assumed by the analytic models. However, the employed analytical models represented a quick investigation tool for a preliminary evaluation of the radiation efficiency of the structure with simply supported boundaries, which perfectly matched the FE results. If required, it was possible to obtain a higher accuracy by means of numerical approaches, taking into account the real fixing condition of the plate’s edges, when these could be modeled.
